# Preclinical Drug-Response Profiling Identifies BMI1 Inhibition as a Therapeutic Option for Hepatoblastoma

**DOI:** 10.3390/ijms27125237

**Published:** 2026-06-10

**Authors:** Salih Demir, Marie Friederike Bentrop, Alina Hotes, Tanja Schmid, Emilie Indersie, Sophie Branchereau, Christian Vokuhl, Beate Häberle, Irene Schmid, Stefano Cairo, Roland Kappler

**Affiliations:** 1 Department of Pediatric Surgery, Dr. von Hauner Children’s Hospital, University Hospital, LMU Munich, Lindwurmstreet 2a, 80337 Munich, Germany; 2 XenTech, 91000 Evry, France; 3 Bicêtre Hospital, AP-HP Paris Saclay University, 94270 Le Kremlin-Bicêtre, France; 4 Institute of Pathology, University Hospital Bonn, 53127 Bonn, Germany; 5 Department of Pediatrics, Dr. von Hauner Children’s Hospital, University Hospital, LMU Munich, 80337 Munich, Germany; 6 Champions Oncology, Rockville, MD 20850, USA

**Keywords:** hepatoblastoma, polycomb repressive complex, BMI1, PTC596, apoptosis

## Abstract

Hepatoblastoma (HB), the most common pediatric liver cancer, exhibits marked variability in therapeutic response despite minimal genetic heterogeneity, implicating epigenetic regulation as a key driver of tumor behavior. Among these, polycomb repressor complexes (PRC) remain poorly explored as therapeutic targets. Integrative analysis of samples from patients with HB and public datasets identified BMI1, a core component of PRC1, as significantly upregulated, with high expression strongly associated with aggressive disease and poor survival. Functional screening of epigenetic inhibitors across 15 HB cell lines revealed BMI1 inhibition as the most effective therapeutic strategy, with strong concordance between in vitro predictions and in vivo responses in patient-derived xenograft (PDX) models. The BMI1 inhibitor PTC596 demonstrated the highest potency, consistently suppressing tumor growth across models. Mechanistically, PTC596 induced BMI1 degradation, reduced histone H2A ubiquitination, impaired microtubule dynamics, and restored intrinsic apoptosis by shifting the BCL2–BAX balance, leading to caspase-3/7 activation. Transcriptomic profiling confirmed apoptosis as the most significantly enriched pathway. In vivo, PTC596 markedly reduced tumor burden and proliferation while inducing pro-apoptotic signaling, without detectable toxicity. Together, these findings establish BMI1 as a critical oncogenic dependency in HB, demonstrate the value of robust preclinical tumor modeling for therapeutic validation, and identify PTC596 as a promising, mechanism-based treatment strategy.

## 1. Introduction

Clinical trials remain among the most complex and costly stages of drug development, and despite substantial advances in oncology, the failure rate of candidate therapeutics in clinical trials is still unacceptably high [1]. A key contributor to these failures is suboptimal target selection and insufficient understanding of which patient populations are most likely to benefit from a given therapeutic intervention. Robust pre-clinical data that enables patient stratification can markedly increase the likelihood of clinical success by ensuring that only those patients whose tumors harbor the relevant molecular features are enrolled. Consequently, pre-clinical biological models that faithfully capture both the genomic and phenotypic complexity of cancer, as well as the diversity of therapeutic responses, are essential for de-risking early-stage development [2]. In this context, immortalized cancer cell lines and patient-derived xenografts (PDX) remain essential, and when deployed at scale, are particularly powerful for identifying drug-response markers and enabling high-throughput screening [3].

Over the past decade, cancer genomics efforts have dramatically transformed our understanding of the molecular alterations that drive hepatoblastoma (HB), the most common primary malignant liver tumor in children [4,5,6,7,8]. Despite substantial variability in clinical behavior [9,10], HB is characterized by an exceptionally low mutational burden, harboring an average of only three somatic mutations per tumor genome [5,8]. Among these, activating mutations in the *CTNNB1* (*ß-catenin*) gene are the most frequent, occurring in approximately 70% of sporadic cases and leading to constitutive activation of the Wnt/β-catenin signaling pathway, a key oncogenic driver in HB pathogenesis [11,12]. Other mutations occur in the *NFE2L2* (*nuclear factor erythroid 2-like 2*) and *ARID1A* (*AT-rich interaction domain 1A*) genes, as well as in the *TERT* (*telomerase reverse transcriptase*) promoter, although they are found at low frequencies of around 5% [5,6,7,8]. Beyond genetic alterations, accumulating evidence highlights the pivotal role of epigenetic mechanisms in HB development and progression [4,6,7]. In particular, histone modifications and DNA methylation have been implicated in shaping tumor heterogeneity and influencing patient prognosis [13,14].

A central component of epigenetic regulation is provided by the polycomb repressive complexes (PRCs), evolutionarily conserved multiprotein assemblies that mediate transcriptional silencing through chromatin remodeling [15]. PRC2 initiates gene repression by catalyzing trimethylation of histone H3 at lysine 27 (H3K27me3), a histone mark associated with transcriptional silencing [16]. This modification subsequently facilitates recruitment of PRC1, which monoubiquitinates histone H2A at lysine 119 (H2AK119ub), reinforcing a repressive chromatin state [17]. While components of the PRC2 complex have been most closely linked to various cancers, accumulating evidence also highlights an important contribution of PRC1 components to tumorigenesis [18]. In particular, its involvement in the transcriptional repression of pro-apoptotic and anti-proliferative target genes has been extensively documented in numerous studies [18]. However, how these molecular features translate into therapeutic approaches in HB remains poorly understood.

In this study, we aimed to establish a holistic strategy that integrates a comprehensive PDX-based drug testing pipeline, transcriptomic profiling, and extensive in vitro and in vivo validation tests to evaluate PRC1- and PRC2-targeting small molecules as novel therapeutic candidates for HB in the preclinical setting.

## 2. Results

### 2.1. High BMI1 Expression Associates with Aggressive Disease and Reduced Patient Survival

**Figure 1:**
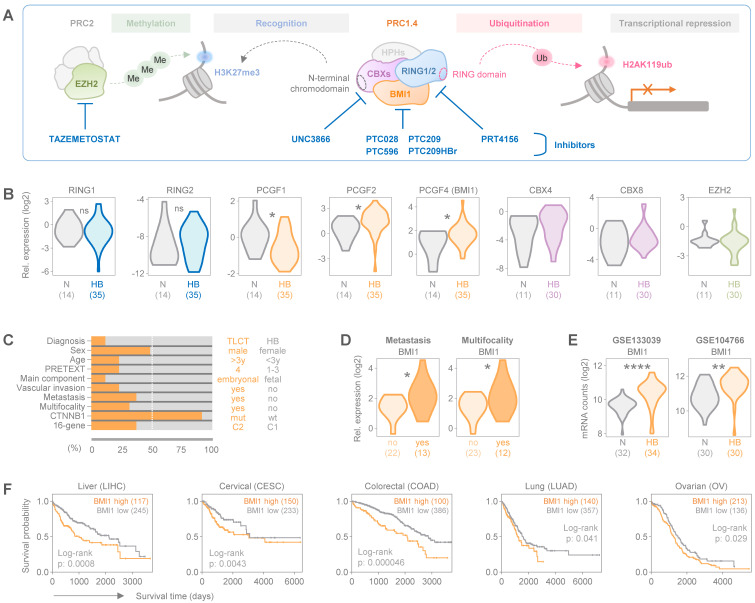
High *BMI1* expression is associated with a poor prognosis in liver cancer. (**A**) Schematic illustration of the polycomb repressive complexes PRC1.4 and PRC2 mediating transcriptional repression, with core proteins of the chromobox protein homolog genes (CBX), polycomb group ring finger (PCGF), and ring finger (RING) families, and the histone marks monoubiquitinylated histone H2A at lysine 119 (H2AK119ub) and trimethylated histone H3 at lysine 27 (H3K27me3). Inhibitors targeting distinct components of polycomb repressive complexes are highlighted in blue. (**B**) Relative RNA expression levels of specific PRC1 and PRC2 components in hepatoblastoma (HB) and normal liver (N) samples, quantified by RT-PCR and normalized to the housekeeping gene *TBP*. Sample numbers are indicated in brackets. (**C**) Horizontal stacked bar plots displaying clinical features of 35 patients with HB and transitional liver cell tumor (TLCT). (**D**) Violin plots displaying relative *BMI1* expression stratified by the high-risk features metastasis, multifocality, and outcome. (**E**) RNA expression levels of the *BMI1* gene in normal liver (N) and HB from datasets GSE133039 and GSE104766. Gene expression values are presented as fragments per kilobase of transcript per million (FPKM) mapped reads. (**F**) Kaplan–Meier survival curves for patients with liver (*n* = 362), cervical (*n* = 383), colorectal (*n* = 486), lung (*n* = 497), and ovarian (*n* = 349) cancer, retrieved from the Human Protein Atlas database and stratified by high or low *BMI1* expression. The log-rank Mantel-Cox test was used to calculate significance. For all violin plots, horizontal lines indicate median expression and student’s *t*-test was applied for statistical calculations. * *p* < 0.05, ** *p* < 0.01, **** *p* < 0.0001 were considered significant and “ns” represents no significance.

The initiation and progression of cancer largely depend on dysregulated polycomb repressive complexes [18], with PRC2 initiating gene repression by catalyzing trimethylation of histone H3 at lysine 27 (H3K27me3), which in turn recruits PRC1 to monoubiquitinate histone H2A at lysine 119 (H2AK119ub) (Figure 1A). To examine the role of core components of PRCs in HB, we isolated tumor RNA from a cohort of 35 patients with HB and quantified the mRNA levels of enhancer of zeste homolog 2 (*EZH2*), the chromobox protein homolog genes *CBX4* and *CBX8*, the ring finger genes *RING1* and *RING2*, as well as the polycomb group ring finger genes *PCGF1*, *PCGF2*, and *PCGF4* (alias *BMI1*) using quantitative real-time PCR. Among these genes, only *PCGF2* and *BMI1* were markedly upregulated in HB samples compared with 14 adjacent normal liver samples (Figure 1B).

Our HB cohort encompassed all clinical and molecular subtypes, including high-risk features such as age older than 3 years (23%), transitional liver cell tumors (11%), embryonal histology (11%), large PRETEXT 4 tumors (23%), multifocality (23%), metastasis (37%), vascular invasion (23%), *CTNNB1* mutation (91%), and the C2 subtype of the 16-gene signature classification (Figure 1C). Association analyses revealed that, among PRC components, only elevated *BMI1* expression was strongly associated with high-risk clinical features such as metastasis and multifocality, and was more importantly linked to poor patient outcomes, highlighting its clinical relevance as a therapeutic target. (Figure 1D). Analysis of two independent, comprehensive gene expression datasets of patients with HB further confirmed the significantly elevated *BMI1* levels in patients with HB (Figure 1E).

To explore the clinical relevance of these findings, we assessed survival outcomes of patients with cancer stratified by high versus low *BMI1* expression. As survival data were not available for patients with HB, we extended our analysis to several other cancer types included in the Human Protein Atlas database. Kaplan–Meier survival curves demonstrated that patients with cancer with high *BMI1* expression consistently had significantly poorer 5-year survival than those with low expression, including liver cancer (Figure 1F).

Collectively, these results demonstrate the association of high *BMI1* expression with HB, clinical high-risk features, and inferior survival outcomes, supporting its potential role as a promising target for therapeutic intervention.

### 2.2. BMI1 Inhibitors Are Effective in Hepatoblastoma

**Figure 2:**
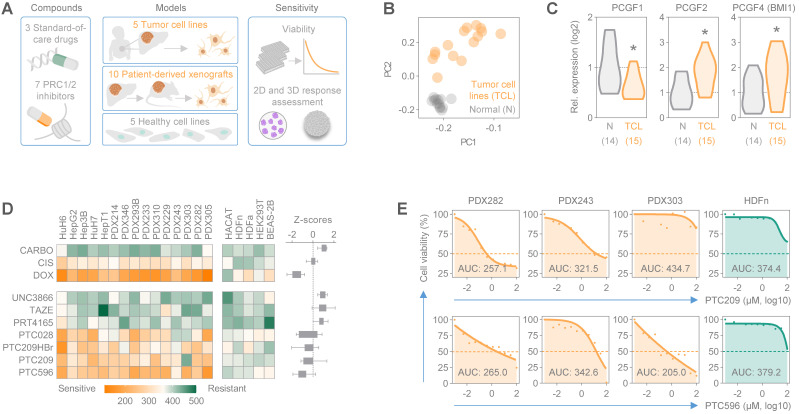
BMI1 inhibition effectively reduces the growth of liver cancer models. (**A**) Schematic overview of the in vitro drug testing platform comprising five publicly available hepatoblastoma cell lines and ten patient-derived xenograft (PDX) lines. (**B**) Principal component analysis of expression data for the eight PRC1 and PRC2 component genes across all cell lines. (**C**) Relative RNA expression levels of the polycomb group ring finger (PCGF) genes in the cell lines included in the platform, quantified by RT-PCR and normalized to the housekeeping gene *TBP*. Dashed line indicates the mean expression level of normal liver tissue and the horizontal lines in violin plots indicate median expression values. (**D**) Heatmap showing drug sensitivity of 15 liver tumor (left) and five non-cancerous control (middle) cell lines to the three standard-of-care therapy drugs, carboplatin (CARBO), cisplatin (CIS), and doxorubicin (DOX), as well as the seven epigenetic perturbagens, UNC3866, tazemetostat (TAZE), PRT4165, PTC028, PTC209-HBr, PTC209, and PTC596. The heatmap represents mean area under the curve (AUC) values from two independent cell viability experiments after 48 h treatment, each performed in duplicate (left). The color gradient from green to orange indicates increasing drug sensitivity. Box-and-whisker plots indicate z-scores, reflecting overall sensitivity towards indicated drugs (right). The dashed line marks the mean z-score of cisplatin. (**E**) Individual dose–response curves for three HB PDX models and neonatal fibroblasts (HDFn) upon exposure to ten increasing concentrations (0.01–100 µM) of PTC209 and PTC596. Data points represent means from two independent experiments performed in duplicate. *p*-value was calculated using a two-tailed unpaired Student’s *t*-test, with significance defined as * *p* < 0.05.

Given their roles in cancer, several small molecules targeting PRC components have been developed, including inhibitors of BMI1 (PTC028, PTC209, PTC209-HBr, and PTC596), EZH2 (Tazemetostat), the interaction of CBX7 with histone H3 (UNC3866), and BMI1–RING1 interaction (PRT4165) (Figure 1A). To evaluate the tumor growth-inhibiting potential of these compounds in HB, we used our in vitro drug testing platform, which comprises five classical liver cancer models, ten PDX-derived cell lines, and five non-cancerous controls [19] (Figure 2A). Principal component analysis of the expression data for the eight PRC1 and PRC2 component genes measured in primary cases across all cell lines showed that this minimal gene set is sufficient to distinguish the 15 models of the platform from normal liver tissue, underscoring the importance of epigenetic regulator in HB (Figure 2B). Notably, the three PCGF genes that were significantly differentially expressed in primary HB exhibited the same expression pattern in the models, highlighting their ability to closely recapitulate patients’ tumors (Figure 2C). Among the compounds tested, the four BMI1 inhibitors proved the most potent, effectively suppressing HB growth compared to EZH2, CBX, and RING inhibitors, as evidenced by lower area-under-the-curve (AUC) values and markedly reduced z-scores (Figure 2D). The first-generation BMI1 inhibitor PTC209 [20] was the second most effective compound, showing responses in most models, but notably not in PDX303 (Figure 2D). Strikingly, all liver cancer models exhibited pronounced sensitivity to the next-generation BMI1 inhibitor PTC596 [21], which was almost as effective as doxorubicin, the standard therapy for patients with high-risk HB (Figure 2D). Moreover, all non-cancerous control samples exhibited limited responsiveness to BMI1 inhibition, underscoring its selective activity and favorable safety profile as a potential therapeutic strategy for HB. Individual growth curves of PTC209- and PTC596-treated sensitive and non-responsive tumor models and neonatal fibroblasts illustrate the spectrum of responses to BMI1 inhibition (Figure 2E).

### 2.3. Translating Screening Predictions into Preclinical Therapeutic Validation

**Figure 3:**
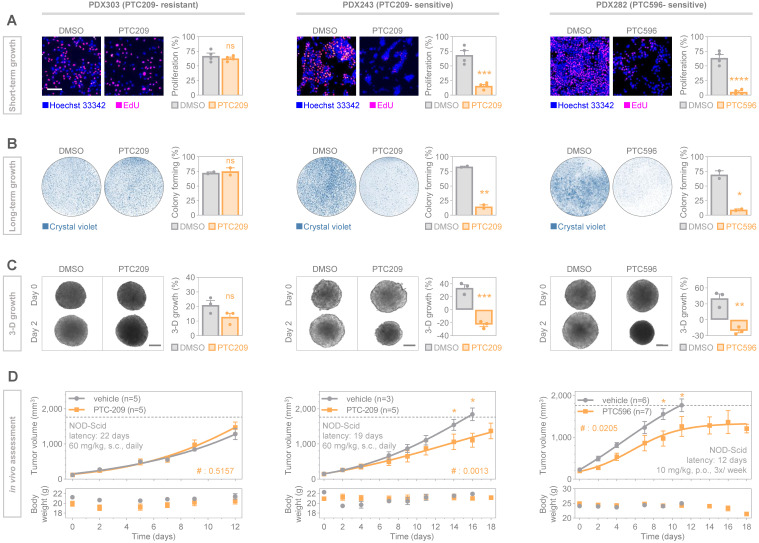
In vitro drug testing predicts in vivo response to BMI1 inhibition. PDX303 (left panel), PDX243 (middle panel), and PDX282 (right panel) HB models following 250 nM PTC209, 250 nM PTC596, or DMSO exposure. (**A**) Immunofluorescent images of EdU-stained proliferating cells (magenta). Proliferation rates were calculated in relation to Hoechst 33342-stained nuclei (blue) after 24 h of treatment. (**B**) Clonogenic potential was detected by crystal violet-stained colony formation after 10 days of treatment. (**C**) Three-dimensional growth of established HB spheroids after 48 h treatment, given as brightfield images. (**D**) Tumor growth in mice with experimental conditions (top) and body weight measurements (bottom) for the PDX303, PDX243, and PDX282 models treated with PTC209, PTC596, or vehicle. Numbers in brackets specify animal numbers, symbols represent mean tumor volumes and body weights ± SEM, and dashed lines indicate maximum permissible values. For all graphs, student’s *t*-test was applied for statistical calculations and the significance is given as: ns, not significant, * *p* < 0.05, ** *p* < 0.01, *** *p* < 0.001, **** *p* < 0.0001. Curve comparisons were determined by applying a mixed-effects model (two-way ANOVA). # denotes the *p* value and *p* < 0.05 is considered significant. All scale bars are adjusted to 100 µm.

To evaluate whether the drug-response predictions generated on our in vitro drug testing platform accurately reflect the in vitro and in vivo responses, we further investigated the effects of the BMI1 inhibitors PTC209 and PTC596 in three PDX models: the predicted PTC209-resistant PDX303, the predicted PTC209-sensitive PDX243, and the PTC596-sensitive PDX282. As anticipated, both predicted-sensitive models exhibited marked growth suppression in short- and long-term assays, as evidenced by reduced levels of newly synthesized EdU-positive DNA (Figure 3A) and diminished colony-forming capacity (Figure 3B). In contrast, PDX303 showed no significant response to PTC209, corroborating its classification as PTC209-resistant (Figure 3A,B). Using established HB spheroid cultures, we further demonstrated that PTC209 and PTC596 effectively inhibited the three-dimensional growth of the sensitive models, as indicated by a substantial reduction in spheroid volume, whereas PDX303 spheroids remained largely unaffected compared with vehicle-treated controls (Figure 3C). To also evaluate the selectivity in vivo, immunocompromised mice bearing subcutaneously propagated tumors of the respective PDX models were treated after an approximately three-week latency period with either subcutaneously injected PTC209 (60 mg/kg body weight according to [20]), orally administered PTC596 (10 mg/kg body weight according to [21]), or vehicle. We observed a significant reduction in tumor volume in PDX243 and PDX282 models compared to vehicle-treated controls, whereas PDX303 again showed no response, consistent with our in vitro findings (Figure 3D). Importantly, body weight remained stable during the treatment period, and no adverse physical or behavioral changes were noted, indicating that the regimens were well tolerated. In summary, these data demonstrate that PTC209 and PTC596 disrupt tumor growth exclusively in HB models identified as sensitive by our in vitro drug screening platform, while the resistant model displayed no measurable response. This validates the predictive power of our pipeline for anticipating in vivo therapeutic efficacy and underscores its value for guiding preclinical treatment strategies.

### 2.4. BMI1 Inhibition by PTC596 Reactivates BAX-Mediated Apoptosis in Hepatoblastoma

**Figure 4:**
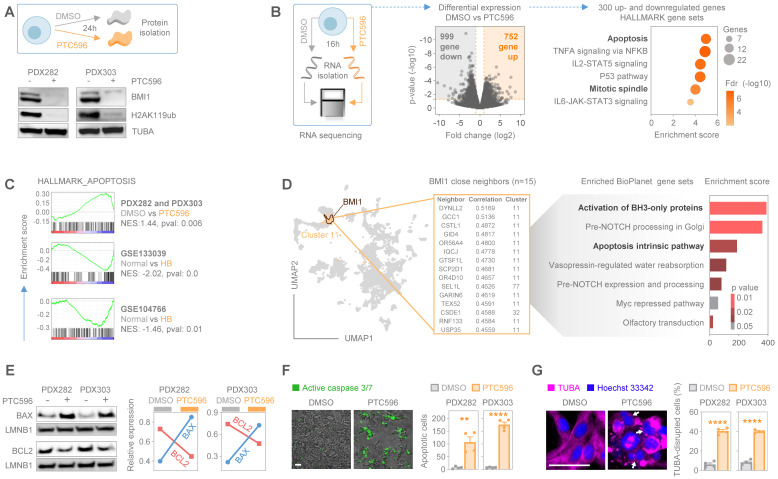
PTC596 induces cell death by increasing the BAX-BCL2 ratio. (**A**) Western blot analysis of PDX282 and PDX303 cells showing downregulation of BMI1 protein levels and mono-ubiquitinated lysine 119 of histone H2A (H2AK119ub) following 24 h incubation with DMSO or 250 nM PTC596. Alpha-tubulin (TUBA) served as loading control. (**B**) Volcano plot of expressed genes generated by RNA sequencing demonstrating commonly up- (orange) and down-regulated (gray) genes in PDX282 and PDX303 after 16 h incubation with DMSO or 250 nM PTC596. Dashed lines represent log2 fold-change and *p* < 0.05 significance thresholds. Bubble plot shows gene set enrichment analysis of the top 300 up- and down-regulated genes. Top-scoring HALLMARK gene sets are listed, with bubble size representing the number of enriched genes, distance from *y*-axis reflecting fold enrichment, and color scale corresponding to false discovery rate (fdr). (**C**) Individual enrichment plots showing the top-ranked HALLMARK_APOPTOSIS term in PTC596-treated (blue) versus DMSO-treated (red) PDX models, as well as in hepatoblastoma (HB) samples (blue) of two independent datasets (GSE133039 and GSE104766) compared to normal liver samples (red). (**D**) UMAP visualization and clustering of single-cell RNA sequencing data from 31 human tissues obtained from the Human Protein Atlas (left). BMI1 and its affiliated cluster are highlighted in orange. The table displays the 15 closest neighbors of BMI1 based on correlation values (middle). The horizontal bar graph summarizes BioPlanet pathway enrichment analysis of these 15 BMI1-neighboring genes (right). (**E**) Western blot analysis (left) and normalized band density quantifications (right) demonstrating nuclear expression levels of BAX (blue) and BCL2 (red) proteins in PDX models treated for 24 h with DMSO or 250 nM PTC596. Lamin B1 (LMNB1) was used as nuclear loading control. (**F**) Apoptosis after 24 h treatment, measured by fluorescent detection of active caspase-3/7 substrates (green). (**G**) Multinucleation (highlighted by arrows) and disorganized microtubule distribution detected by immunofluorescent staining of nuclei (DAPI, blue) and α-tubulin (TUBA) in HB cells exposed to 250 nM PTC596 for 24 h. All bar graphs represent the mean ± SEM, and symbols indicate the individual measurements. Statistical significance was determined by two-tailed unpaired Student’s *t*-test: ** *p* < 0.01, **** *p*< 0.0001. All scale bars are adjusted to 50 µm.

Since the second-generation BMI1 inhibitor PTC596 proved to be the most effective compound in our preclinical testing and is currently undergoing clinical evaluation in other cancers [22,23], we wanted to investigate the molecular responses it triggers in HB. PCT596 has been described to directly inhibit BMI1 in acute myeloid leukemia by promoting its downregulation on the protein level, thereby reducing downstream H2AK119 ubiquitination [21]. Consistent with this mechanism, we observed clear BMI1 protein degradation by Western blot analysis, accompanied by marked inhibition of H2AK119 ubiquitination following PTC596 treatment (Figure 4A). After confirming on-target BMI1 inhibition by PTC596, we next investigated the transcriptomic consequences of BMI1 inhibition by performing RNA sequencing on two PTC596-responsive PDX models. Differential expression analysis revealed 999 downregulated and 752 upregulated genes (Figure 4B). To understand the molecular importance of these transcriptional changes, we conducted pathway enrichment analysis utilizing the top 300 up- and down-regulated genes. “HALLMARK_APOPTOSIS” emerged as the most significantly enriched pathway (Figure 4B), as supported by the corresponding enrichment plot (Figure 4C), suggesting that PTC596 likely restores dysregulated apoptotic pathways in HB. Consistent with this, analysis of two independent HB expression datasets showed an inverse enrichment of apoptosis in tumor samples, highlighting suppression of apoptotic signaling as a key hallmark of HB (Figure 4C).

To further investigate the role of BMI1 in the regulation of apoptosis, we analyzed publicly available single-cell RNA sequencing data comprising a transcriptomic atlas of 31 healthy tissues and organs, encompassing 557 distinct cell type clusters. This approach aimed to identify genes consistently co-expressed with *BMI1* across physiologically relevant cellular contexts and to determine whether these associated genes converge on specific biological pathways. Correlation analysis identified 15 genes in close proximity to BMI1 across multiple proliferative and progenitor-like cellular populations represented in the dataset. Subsequent pathway enrichment analysis of these neighbors revealed “activation of BH3-only proteins” as the most significant term, suggesting that BMI1 may influence apoptosis through modulation of BH3-only protein dynamics (Figure 4D). BH3-only proteins, a subgroup of the BCL2 family, are key mediators of pro-apoptotic signaling within the tightly regulated BCL2 family network that balances cell survival and death in response to physiological and pathological stimuli [24]. Consistent with this mechanism, we observed increased protein levels of the pro-apoptotic effector BAX and reduced expression of the anti-apoptotic inducer BCL2 following 24 h of PTC596 treatment (Figure 4E). Consequently, PTC596 induced apoptosis in PDX282 and PDX303 cells, as evidenced by elevated levels of active caspase-3/7 substrates in treated HB cells (Figure 4F).

In addition to apoptosis, pathway enrichment analysis also identified the “HALLMARK_MITOTIC_SPINDLE” signature as significantly enriched (Figure 4B). Interestingly, PTC596 has previously been reported to inhibit tubulin polymerization in cell-free assays and to downregulate tubulin-related genes [25]. Therefore, we further investigated the effect of PTC596 on intracellular microtubule organization by immunostaining. As shown in Figure 4G, vehicle-treated control cells displayed well-organized microtubule networks, whereas PTC596-treated cells exhibited multinucleation and markedly disorganized microtubule distribution. These findings suggest that PTC596 disrupts intracellular microtubule assembly in HB cells, a process essential for mitotic spindle formation and accurate segregation of chromosomes during mitosis.

Collectively, our data demonstrate that pharmacological inhibition of BMI1 by PTC596 shifts the balance from anti-apoptotic to pro-apoptotic proteins and disrupts microtubule dynamics, thereby activating the intrinsic apoptotic pathway.

### 2.5. Pro- and Anti-Apoptotic Protein Shift Indicates Response to PTC596 In Vivo

**Figure 5:**
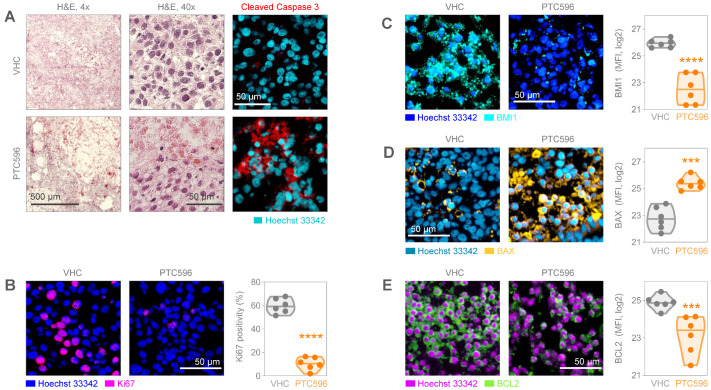
Mechanism of action of PTC596 treatment in vivo. (**A**) Hematoxylin/eosin (H&E) staining of vehicle (VHC) and PTC596-treated tumors (left and middle panels), and corresponding immunofluorescent staining of cleaved caspase-3 (red), indicating apoptotic regions, counterstained with Hoechst 33342 (blue) (right panel). (**B**) Representative immunofluorescent images of Ki67 staining in vehicle (VHC) and PTC596-treated tumors. Proliferation was quantified as the ratio of Ki67-positive nuclei to Hoechst 33342-stained nuclei in two representative areas from three tumors per condition. (**C**–**E**) Intracellular levels of (**C**) BMI1, (**D**) BAX, and (**E**) BCL2 proteins in vehicle (VHC)- and PTC596-treated tumor sections, shown by immunofluorescent staining relative to Hoechst 33342-stained nuclei. Mean fluorescent intensities (MFI) values represent BMI1 expression in two representative areas from three tumors per condition. All bar graphs display mean ± SEM, and symbols indicate the individual areas. *p*-values were calculated using a two-tailed unpaired Student’s *t*-test, with significance defined as *** *p* < 0.001, **** *p* < 0.0001.

To assess whether the identified molecular mechanism of action, specifically the rebalance of proteins of the BCL2 family, could serve as a biomarker of PTC596 response in vivo, we performed histological and immunofluorescent analyses of tumor specimens after treatment. PTC596-treated tumors displayed recurrent areas of eosinophilic remnants and condensed nuclei, indicative of cell death and tissue disruption (Figure 5A, left and middle panels). These regions were positive for cleaved caspase-3, confirming induction of apoptosis (Figure 5A, right panel). In addition, immunofluorescent analysis revealed a markedly reduced number of Ki67-positive proliferating cells in PTC596-treated tumors relative to controls (Figure 5B). This was accompanied by a substantial degradation of BMI1 in tumors from PTC596-treated mice, whereas high BMI1 expression persisted in vehicle-treated controls (Figure 5C). More importantly, we also detected a pronounced induction of the pro-apoptotic protein BAX and a concomitant downregulation of the anti-apoptotic protein BCL2 in vivo (Figure 5D,E).

Overall, our data clearly demonstrate that PTC596 exerts potent antitumor activity in vivo by shifting BCL2-family protein dynamics toward a pro-apoptotic state and underscore the strong translational potential of BAX protein monitoring as a candidate pharmacodynamic marker.

## 3. Discussion

Genetic alterations alone cannot fully explain the clinical heterogeneity observed in “genetically simple” hepatoblastoma (HB) [5,8]. Multiple studies have proposed that broad transcriptional changes, largely driven by epigenetic regulatory systems, are fundamental contributors to this diversity [4,6,7]. Among the major epigenetic regulators, the polycomb repressive complexes (PRCs) have emerged as pivotal orchestrators of transcriptional control in cancer, capable of silencing tumor suppressive networks and thereby shaping disease behavior and therapeutic response [15]. Through a comprehensive drug testing platform for pediatric liver cancers, followed by in vitro and in vivo validation, we identified the PRC1 inhibitor PTC596 as a promising therapeutic candidate for HB. PTC596 functions through inhibiting the PRC1 subunit BMI1, leading to modulation of BCL2 family proteins that promote apoptosis, while maintaining a favorable safety profile in vivo.

Our transcriptional analysis identified *BMI1* as the most prominently upregulated gene in HB specimens compared with normal liver tissue, standing out relative to other components of PRC1 and PRC2. BMI1, the defining PCGF protein within the canonical PRC1.4 complex [18], is a well-established oncogenic driver implicated in the development of numerous cancers [26], including hepatocellular carcinoma [27]. Across a wide range of malignancies, including hematological malignancies and liver cancer, aberrant *BMI1* overexpression is consistently associated with advanced disease stage, aggressive clinicopathological features, therapeutic resistance, and poor patient survival [28,29]. In line with these reports, our data show that increased *BMI1* abundance in HB strongly associates with multifocal tumor growth and metastasis, underscoring its potential as an indicator of high-risk disease. Our survival analyses across multiple cancer types further demonstrate significantly worse outcomes in patients with high *BMI1* expression, reinforcing its role in driving aggressive tumor biology. Notably, prior studies have also linked high *BMI1* expression to inferior survival in liver cancer specifically [30], likely reflecting its essential function in sustaining cancer stem cells, a subpopulation critical for tumor initiation, therapy resistance, and recurrence [31]. In line with this, recent evidence suggests that *Bmi1* functions as an oncogene in mice by promoting hepatic progenitor cell proliferation and sustaining AKT-mediated liver tumor development [32], a signaling pathway that is also highly relevant in HB pathogenesis [14]. Collectively, these observations firmly establish BMI1 as a clinically relevant oncogenic factor in HB, providing a strong rationale for targeting its expression or downstream regulatory networks as a therapeutic strategy [33].

Several small-molecule inhibitors, including PTC209 and the second-generation compound PTC596, have been developed to selectively target BMI1, with their antitumor efficacy extensively evaluated in preclinical models [25,34,35,36]. PTC209, although effective in reducing the growth of BMI1-dependent colorectal cancer cells by impairing tumor-initiating cell function [20], was ultimately not advanced clinically due to poor pharmacokinetic properties [21]. To overcome these limitations, PTC596 was developed with enhanced cell permeability, improved potency, and the ability to promote BMI1 degradation at nanomolar concentrations [21]. PTC596 also exhibits high oral bioavailability and can cross the blood–brain barrier, making it suitable for treating a broad range of malignancies [36]. Importantly, PTC596 has progressed to early-phase clinical trials for advanced solid tumors, including glioblastoma and colorectal cancer, where it has shown a favorable safety profile with manageable gastrointestinal toxicity [22,23]. In our study, PTC596 consistently outperformed PTC209, producing robust antitumor responses across all 15 liver tumor models in our in vitro testing platform, as reflected by low IC50 values and favorable z-scores. In addition, PTC596 markedly reduced tumor cell growth in both adherent culture conditions and three-dimensional tumor spheroids, aligning with observations reported in many other cancers [25,34,35,36]. Consistent with its preclinical record in other cancers, PTC596 showed no safety concerns in our in vivo experiments. As inhibitors targeting other PRC subunits investigated in our study did not achieve significant antitumor activity, our results strongly support PTC596 as a promising therapeutic option for patients with HB.

Mechanistically, PTC596 inhibits BMI1 function by accelerating its protein degradation, thereby reducing downstream H2AK119 ubiquitination, a key PRC1-mediated chromatin mark associated with transcriptional repression [21]. Loss of this modification has been shown to reactivate tumor suppressor genes [15]. Consistent with this mechanism, our functional analyses revealed that PTC596 exposure of HB cells induces post-translational degradation of BMI1, decreases H2AK119 ubiquitination, and activates apoptosis-related genes. BMI has previously been reported to repress genes involved in apoptosis in other cancers [37,38,39], and restoring apoptosis is a central therapeutic goal, as malignant cells frequently evade programmed cell death by disrupting intrinsic apoptotic pathways [40]. This mechanism is primarily governed by the proteins of the BCL2 family, which balance pro- and anti-apoptotic signals in response to cellular stress. Pro-apoptotic members such as BAX and BAK promote mitochondrial dysfunction and caspase 3 activation, whereas anti-apoptotic proteins preserve mitochondrial integrity and prevent cytochrome c efflux [40]. Consequently, the BAX/BCL2 ratio acts as a critical molecular switch for intrinsic apoptotic activation [40]. Consistent with this model, PTC596 increased the BAX/BCL2 protein ratio in HB cells, followed by caspase-3 cleavage, indicating restoration of the intrinsic apoptosis and subsequent tumor cell death. Immunostaining of tumor samples from PTC596-treated mice likewise confirmed an elevated BAX/BCL2 ratio in vivo, reinforcing the activation of apoptosis in a preclinical setting. These findings are consistent with previous studies reporting that PTC596 downregulates anti-apoptotic proteins [21,39]. Interestingly, BMI1 itself has been shown to broadly modulate the transcription of pro- and anti-apoptotic genes [41]. Indeed, BMI1 knockdown induces BAX expression and enhances apoptosis in breast cancer [42] and neuroblastoma [43], mirroring the effects observed with PTC596 in our study. Given that BCL2 family proteins act as sensors of cellular stress [40], monitoring their dynamics may serve as a useful surrogate marker of tumor response to BMI1-targeted therapy. Taken together, these findings indicate that BMI1 inhibition by PTC596 shifts the balance of BCL2 family proteins toward apoptosis by increasing the BAX/BCL2 ratio, ultimately triggering initiation of apoptosis in HB.

In addition to its direct pro-apoptotic effects, we identified disruption of microtubule dynamics as a secondary mechanism of PTC596, consistent with previous findings on pancreatic ductal adenocarcinoma [25]. In our study, PTC596 treatment significantly impaired intracellular microtubule organization and induced multinucleation in HB cells. The increased sensitivity of PDX models to PTC596, but not PTC209, most likely reflects differences in potency and mechanism of action between the two compounds, rather than intrinsic BMI1 dependency alone. Together, these findings suggest that the pronounced antitumoral effects of PTC596 are likely mediated by both a shift from anti-apoptotic to pro-apoptotic signaling and disruption of microtubule dynamics.

## 4. Materials and Methods

### 4.1. Patients with Liver Cancer

HB tumor and corresponding normal liver tissue samples were collected from patients who underwent surgical resection at the Department of Pediatric Surgery, Dr. von Hauner Children’s Hospital. The study was approved by the Ethics Committee of Ludwig Maximilian University (application number 431-11), and informed consent was obtained from all patients or their legal guardians.

Comparative expression analyses were conducted using mRNA count data from the publicly available datasets GSE133039 [4] and GSE104766 [44], retrieved via the R2 Genomics Analysis and Visualization Platform (http://r2.amc.nl accessed on 14 February 2025).

Transcriptomic and survival data from patients with liver cancer (LIHC, *n* = 362), cervical cancer (CESC, *n* = 383), colorectal cancer (COAD, *n* = 486), lung adenocarcinoma (LUAD, *n* = 497), and ovarian cancer (OV, *n* = 349) were obtained from the Human Protein Atlas (HPA) database v24.0 (https://www.proteinatlas.org/ accessed on 14 February 2025), which integrates TCGA RNA-seq data of patients with cancer. These data were used for survival analyses.

Publicly accessible single-cell RNA sequencing (scRNA-seq) data and cell cluster visualizations were also retrieved from HPA v24.0 accessed on 14 May 2025 [45]. Genes detected as close neighbors of *BMI1* by correlation analysis were subjected to BioPlanet pathway enrichment analysis by means of the Enrichr web tool (https://maayanlab.cloud/Enrichr/ accessed on 21 March 2025).

### 4.2. Quantitative Real-Time PCR

RNA isolation, cDNA synthesis, and quantitative real-time PCR using a SYBR green-based detection system (Bio-Rad Laboratories, Hercules, CA, USA) were performed as described previously [46]; PCR amplifications in duplicates were carried out on a Mastercycler RealPlex2 instrument (Eppendorf, Hamburg, Germany) and data were acquired using the realplex v2.2 software. Relative gene expression levels were calculated using the 2^−ΔΔCt^ method [47] and normalized to the expression of the TATA-box binding protein (*TBP*) gene as internal control. The primers (5′->3′ orientation) used were: RING1, AGGTGGAGAAATTGAGCTCGT, TCTTCACATACCTCGTCTGGC; RING2, CGGGGTTCCAGTGTAGAGG, AGCCTGAGACATTGGGTTTTAG; PCGF1, CAGCCTTTAAGATGGCGTCTC, AACCTCCTCCTCGTTCCGTA; PCGF2, AAAGAGAAAACAGGGGTGCG, TACTTGCTGGGCACATCCAT; PCGF4 (BMI1), GCTGGTTGCCCATTGACAG, AAAAATCCCGGAAAGAGCAGC; CBX4, TGATCGCCTTCCAGAACAGG, AAAGGTAGGCACCTGCACC; CBX8, GCGAGTCAACATGGAGCTTT, TCCATGCGTCCTTTCCGTAT; EZH2, AACAGCGAAGGATACAGCCTG, TTGTGTTGGAAAATCCAAGTCACT; and TBP, GCCCGAAACGCCGAATAT, CCGTGGTTCGTGGCTCTCT. Principal component analysis was performed using ClustVis v2.0 [48].

### 4.3. Drug Testing Platform and Viability Assay

The in vitro drug testing platform consists of three HB cell lines (HuH6, HepG2, HepT1), two HCC cell lines (Hep3B, HuH7), in addition to 10 HB PDX tumor cell lines (PDX214, PDX229, PDX233, PDX243, PDX282, PDX293B, PDX303, PDX305, PDX310, PDX346). Moreover, epidermal keratinocytes (HaCaT), primary neonatal (HDFn) and adult (HDFa) human dermal fibroblasts, embryonal kidney epithelial cells (HEK293T) as well as human bronchial epithelial cells (BEAS-2B) were included as non-cancerous controls. Detailed information and maintenance of the cell lines and the PDXs are as previously reported [19].

Models in the platform were seeded as 5 × 10^4^ cells in 96-well plates, as two replicates, and then exposed to 10 increasing concentrations of the compound-of-interest, ranging from 5 nM to 100 µM in 1:3 constant dilution ratio, for 48 h. MTT (3-(4, 5-dimethylthiazol-2-yl)-2, 5-diphenyltetrazolium bromide) viability assays were carried out as recommended by the supplier (Carl Roth, Karlsruhe, Germany). Nonlinear regression curves of drug response versus the inhibitors were plotted and the sensitivities were determined by calculating half-maximal inhibitory concentrations (IC50) and area under the curve (AUC) values, using GraphPad Prism 8 (GraphPad Software, San Diego, CA, USA). Z-scores were calculated with the formula: [(individual AUC value) − (mean AUC)]/(standard deviation). The compounds tested were: carboplatin, cisplatin, doxorubicin, PRT4165, PTC028, PTC209, PTC209-HBr, PTC596, tazemetostat, and UNC3866 (all from Selleckchem, Chesterbrook, PA, USA).

### 4.4. Proliferation Assay

Cell proliferation was assessed using the Click-iT EdU Imaging Kit (Thermo Fisher, Waltham, MA, USA) following the manufacturer’s protocol. Briefly, 2 × 10^5^ cells were seeded into 24-well plates and incubated in 100 µM ethynyl-deoxyuridine (EdU) for 24 h. Then, cells were treated with 250 nM PTC209, 250 nM PTC596, or DMSO for an additional 24 h. Subsequently, cells were fixed with 3.7% paraformaldehyde, permeabilized with 0.5% Triton X-100 in PBS, and stained with Hoechst 33342 (Thermo Fisher) along with the Click-iT staining cocktail containing reaction buffer, CuSO_4_, and Alexa Fluor azide (Thermo Fisher). Images were acquired using the EVOS M7000 imaging system (Thermo Fisher). Proliferation was quantified as the ratio of EdU-positive cells to the total number of Hoechst 33342-positive nuclei.

### 4.5. Colony Formation Assay

The long-term proliferative capacity of tumor cells was assessed through a colony formation assay. Briefly, 2 × 10^3^ cells were seeded into 6-well plates and allowed to adhere for 48 h before exposure to 250 nM PTC209, 250 nM PTC596, or DMSO for 10 days. Upon completion of the assay, colonies were fixed and stained with 0.5% crystal violet (Sigma-Aldrich, St. Louis, MO, USA) dissolved in 20% methanol for 2 h. Colony formation was documented using the GelJet Imager (INTAS, Göttingen, Germany) for overall visualization. Colony-forming capability was determined by the percentage of colony-forming units, calculated by dividing the counted cell number by the seeded cell number.

### 4.6. Spheroid Assay

For assessment of three-dimensional growth, 1 × 10^3^ cells suspended in 100 µL per well were seeded into ultra-low attachment, round-bottom 96-well plates (Corning, Corning, NY, USA) and cultured for 5 days to promote spheroid formation. Once spheroids were established, they were exposed to 250 nM PTC209, 250 nM PTC596, or DMSO. Spheroid morphology was documented on day 2 using the EVOS M7000 imaging system (Thermo Fisher). Spheroid volumes at 0 h and 48 h were calculated according to the formula: [(length (µm) × width (µm)^2^)/2].

### 4.7. Animal Studies

The preclinical efficacy studies in mice were performed by Xentech (Evry, France) in compliance with a license for vertebrate animal experimentation granted by the French Ministry of Higher Education, Research and Innovation (APAFIS#29136-2020121415204532). In brief, cryopreserved tumor fragments from PDX243, PDX282 and PDX303 were subcutaneously implanted in the interscapular region of 5-week-old female nude-Foxn1nu mice. Once tumors reached volumes between 75 and 288 mm^3^ after a latency period of 12 to 22 days, mice were treated with vehicle control, 60 mg/kg PTC209 in 10% DMSO/PBS delivered by daily subcutaneous injection, and 10 mg/kg PTC596 administered orally in 0.5% HPMC/0.1% Tween 80 in sterile deionized water using a 3× per week schedule for 3 weeks. Tumor volumes (TV) were calculated using the formula: TV (mm^3^) = [(length (mm) × width (mm)^2^)/2]. Body weights were recorded at each tumor measurement, and animals were monitored daily for general health, behavior, and clinical signs.

Histological examination of liver tumors was performed on cryosections stained with hematoxylin (Carl Roth) and eosin (Sigma-Aldrich) and evaluated by a trained pathologist. For immunofluorescent analyses, cryosections with 5 µm thickness were fixed in 4% paraformaldehyde, permeabilized with 0.5% Triton X-100, and blocked with PBS-T containing 3% BSA and 0.1% glycine. Sections were then incubated overnight with 1:500 primary antibody dilutions against cleaved caspase-3, BMI1, BAX (all from Cell Signaling, Danvers, MA, USA), Ki67 (Abcam, Cambridge, UK), and BCL2 (Antibodies online, Limerick, PA, USA), followed by detection using a 1:1000 Alexa Fluor 555-conjugated anti-rabbit IgG secondary antibody dilution (Thermo Fisher, Waltham, MA, USA) on the following day. All images were captured with the EVOS M7000 (Thermo Fisher) imaging system and mean fluorescence intensity (MFI) values were calculated using ImageJ v1.54p (https://imagej.net/ij/ accessed on 17 March 2025).

### 4.8. RNA Sequencing

Total RNA was isolated from patient-derived xenograft (PDX) cell lines as previously described [49]. RNA libraries were generated from 1 µg of total RNA using the TruSeq Stranded mRNA Library Prep Kit (Illumina, San Diego, CA, USA) and sequenced on the Illumina HiSeq2500 platform as 100 bp paired-end reads, yielding approximately 50 million mapped reads per sample. Sequence alignment to the human reference genome (hg19/GRCh37) with UCSC gene annotations was performed using STAR aligner (v2.4.2a) [50], and gene-level read quantification was carried out with HTSeq-count (v0.6.0) [51]. Read counts were normalized and analyzed for differential gene expression between tumor versus normal tissues or treated versus untreated samples using the Bioconductor package DESeq2 [52].

Top 300 genes that were up- or down-regulated upon PTC596 exposure by more than 2-fold with a *p*-value < 0.05 were analyzed using the ShinyGO v.0.82 (http://bioinformatics.sdstate.edu/go/ accessed on 12 February 2025) database to identify altered pathways based on HALLMARK gene sets of the human molecular signature database (MSigDB v2023.2. Hs).

### 4.9. Western Blot Analysis

Whole cell lysates were isolated and prepared as previously described [53]. For phosphoproteins, cell lysis buffer was supplemented with 1× Halt™ Phosphatase Inhibitor Cocktail (Thermo Fisher, Waltham, MA, USA). Proteins (25 µg) were denatured at 70 °C for 10 min, separated on a 4–20% gradient gel (Thermo Fisher) and transferred to nitrocellulose membranes with 0.2 µm pore size (BioRad, Hercules, CA, USA). The membranes were blocked in 5% milk powder (Carl Roth) diluted in Tris buffer saline with 0.1% Tween 20 (Carl Roth, Karlsruhe, Germany) and probed with corresponding 1:1000 primary antibody dilutions overnight at 4 °C. Following 1 h incubation with the respective 1:2000 HRP-conjugated secondary antibody dilutions, the proteins were detected using enhanced chemiluminescence detection reagent (Amersham Biosciences, Amersham, UK) and imaged with the ChemiDoc XRS+ system (Bio-Rad). The antibodies used were: rabbit-anti-BMI1 [D20B7], rabbit-anti-H2AK119ub [E6C5], rabbit-anti-BAX [E4U1V], rabbit-anti-LMNB1 [D9V6H], anti-mouse HRP, anti-rabbit HRP (all from Cell Signaling Technology, Danvers, MA, USA), mouse-anti-TUBA [DM1A] (Sigma-Aldrich), and rabbit-anti-BCL2 (Antibodies online).

### 4.10. Apoptosis Assay

Apoptosis was detected by CellEvent™ Caspase-3/7 Green Detection Reagent (Thermo Fisher) according to the manufacturer’s instructions, by exposing 2 × 10^5^ cells to 250 nM PTC209, 250 nM PTC596, or DMSO for 24 h and detecting cells that were positive for active substrates of caspase 3 and 7 using the EVOS M7000 imaging system (Thermo Fisher).

### 4.11. Immunofluorescence

First, 2 × 10^5^ cells were seeded onto coverslips one day prior to incubation with either DMSO or PTC596 for 24 h. Following fixation, permeabilization, and blocking, slides were incubated overnight at 4 °C in mouse anti-alpha-tubulin in 5% BSA/PBS in dilutions of 1:2000 (clone DM1A) (Sigma-Aldrich). The next day, slides were incubated with Alexa Fluor 647-conjugated goat anti-rabbit IgG secondary antibody (Invitrogen, Waltham, MA, USA) in a dilution of 1:500 in 5% BSA and 0.1% Triton X-100. Slides were counterstained with 1 µg/mL Hoechst 33342 at room temperature in the dark for 1 h. Images were captured by an EVOS M7000 microscope (Invitrogen) and tubulin-disrupted cells were counted by ImageJ v1.54p (https://imagej.net/ij/ accessed on 26 May 2026).

### 4.12. Statistical Analysis

Statistical analyses were performed using Prism 8.2.0 (GraphPad, La Jolla, CA, USA). The significance of binary variables was determined with a two-tailed, unpaired Student’s *t*-test, and *p* < 0.05 was considered statistically significant. Unless indicated otherwise, values in the figures are presented as means ± standard error of the mean (SEM).

## 5. Conclusions

In conclusion, our findings establish BMI1 as a key oncogenic driver in HB and demonstrate that its selective inhibition by PTC596 induces BMI1 degradation, disrupts PRC1-mediated chromatin regulation, impairs microtubule dynamics, and restores intrinsic apoptotic signaling. By changing the balance of BCL2 family proteins toward apoptosis and activating caspase-3 in vitro and in vivo, PTC596 exhibits robust and broad antitumor efficacy, underscoring its promise as a therapeutic strategy for BMI1-driven liver tumors.

## Data Availability

The datasets used and/or analyzed during the current study are available from the corresponding author on reasonable request.

## References

[1] Dowden H., Munro J. (2019). Trends in clinical success rates and therapeutic focus. Nat. Rev. Drug Discov..

[2] Iorio F., Knijnenburg T.A., Vis D.J., Bignell G.R., Menden M.P., Schubert M., Aben N., Goncalves E., Barthorpe S., Lightfoot H. (2016). A Landscape of Pharmacogenomic Interactions in Cancer. Cell.

[3] Lee J.K., Liu Z., Sa J.K., Shin S., Wang J., Bordyuh M., Cho H.J., Elliott O., Chu T., Choi S.W. (2018). Pharmacogenomic landscape of patient-derived tumor cells informs precision oncology therapy. Nat. Genet..

[4] Carrillo-Reixach J., Torrens L., Simon-Coma M., Royo L., Domingo-Sabat M., Abril-Fornaguera J., Akers N., Sala M., Ragull S., Arnal M. (2020). Epigenetic footprint enables molecular risk stratification of hepatoblastoma with clinical implications. J. Hepatol..

[5] Eichenmüller M., Trippel F., Kreuder M., Beck A., Schwarzmayr T., Häberle B., Cairo S., Leuschner I., von Schweinitz D., Strom T.M. (2014). The genomic landscape of hepatoblastoma and their progenies with HCC-like features. J. Hepatol..

[6] Hirsch T.Z., Pilet J., Morcrette G., Roehrig A., Monteiro B.J.E., Molina L., Bayard Q., Trepo E., Meunier L., Caruso S. (2021). Integrated Genomic Analysis Identifies Driver Genes and Cisplatin-Resistant Progenitor Phenotype in Pediatric Liver Cancer. Cancer Discov..

[7] Nagae G., Yamamoto S., Fujita M., Fujita T., Nonaka A., Umeda T., Fukuda S., Tatsuno K., Maejima K., Hayashi A. (2021). Genetic and epigenetic basis of hepatoblastoma diversity. Nat. Commun..

[8] Sumazin P., Chen Y., Trevino L.R., Sarabia S.F., Hampton O.A., Patel K., Mistretta T.A., Zorman B., Thompson P., Heczey A. (2017). Genomic analysis of hepatoblastoma identifies distinct molecular and prognostic subgroups. Hepatology.

[9] Perilongo G., Maibach R., Shafford E., Brugieres L., Brock P., Morland B., de Camargo B., Zsiros J., Roebuck D., Zimmermann A. (2009). Cisplatin versus cisplatin plus doxorubicin for standard-risk hepatoblastoma. N. Engl. J. Med..

[10] Zsiros J., Brugieres L., Brock P., Roebuck D., Maibach R., Zimmermann A., Childs M., Pariente D., Laithier V., Otte J.B. (2013). Dose-dense cisplatin-based chemotherapy and surgery for children with high-risk hepatoblastoma (SIOPEL-4): A prospective, single-arm, feasibility study. Lancet Oncol..

[11] Koch A., Denkhaus D., Albrecht S., Leuschner I., von Schweinitz D., Pietsch T. (1999). Childhood hepatoblastomas frequently carry a mutated degradation targeting box of the beta-catenin gene. Cancer Res..

[12] Wei Y., Fabre M., Branchereau S., Gauthier F., Perilongo G., Buendia M.A. (2000). Activation of beta-catenin in epithelial and mesenchymal hepatoblastomas. Oncogene.

[13] Claveria-Cabello A., Herranz J.M., Latasa M.U., Arechederra M., Uriarte I., Pineda-Lucena A., Prosper F., Berraondo P., Alonso C., Sangro B. (2023). Identification and experimental validation of druggable epigenetic targets in hepatoblastoma. J. Hepatol..

[14] Demir S., Razizadeh N., Indersie E., Branchereau S., Cairo S., Kappler R. (2024). Targeting G9a/DNMT1 methyltransferase activity impedes IGF2-mediated survival in hepatoblastoma. Hepatol. Commun..

[15] Blackledge N.P., Rose N.R., Klose R.J. (2015). Targeting Polycomb systems to regulate gene expression: Modifications to a complex story. Nat. Rev. Mol. Cell Biol..

[16] Cao R., Wang L., Wang H., Xia L., Erdjument-Bromage H., Tempst P., Jones R.S., Zhang Y. (2002). Role of histone H3 lysine 27 methylation in Polycomb-group silencing. Science.

[17] de Napoles M., Mermoud J.E., Wakao R., Tang Y.A., Endoh M., Appanah R., Nesterova T.B., Silva J., Otte A.P., Vidal M. (2004). Polycomb group proteins Ring1A/B link ubiquitylation of histone H2A to heritable gene silencing and X inactivation. Dev. Cell.

[18] Parreno V., Martinez A.M., Cavalli G. (2022). Mechanisms of Polycomb group protein function in cancer. Cell Res..

[19] Demir S., Hotes A., Schmid T., Cairo S., Indersie E., Pisano C., Hiyama E., Hishiki T., Vokuhl C., Branchereau S. (2024). Drug prioritization identifies panobinostat as a tailored treatment element for patients with metastatic hepatoblastoma. J. Exp. Clin. Cancer Res..

[20] Kreso A., van Galen P., Pedley N.M., Lima-Fernandes E., Frelin C., Davis T., Cao L., Baiazitov R., Du W., Sydorenko N. (2014). Self-renewal as a therapeutic target in human colorectal cancer. Nat. Med..

[21] Nishida Y., Maeda A., Kim M.J., Cao L., Kubota Y., Ishizawa J., AlRawi A., Kato Y., Iwama A., Fujisawa M. (2017). The novel BMI-1 inhibitor PTC596 downregulates MCL-1 and induces p53-independent mitochondrial apoptosis in acute myeloid leukemia progenitor cells. Blood Cancer J..

[22] Jernigan F., Branstrom A., Baird J.D., Cao L., Dali M., Furia B., Kim M.J., O’Keefe K., Kong R., Laskin O.L. (2021). Preclinical and Early Clinical Development of PTC596, a Novel Small-Molecule Tubulin-Binding Agent. Mol. Cancer Ther..

[23] Shapiro G.I., O’Mara E., Laskin O.L., Gao L., Baird J.D., Spiegel R.J., Kaushik D., Weetall M., Colacino J., O’Keefe K. (2021). Pharmacokinetics and Safety of PTC596, a Novel Tubulin-Binding Agent, in Subjects With Advanced Solid Tumors. Clin. Pharmacol. Drug Dev..

[24] Shamas-Din A., Brahmbhatt H., Leber B., Andrews D.W. (2011). BH3-only proteins: Orchestrators of apoptosis. Biochim. Biophys. Acta.

[25] Eberle-Singh J.A., Sagalovskiy I., Maurer H.C., Sastra S.A., Palermo C.F., Decker A.R., Kim M.J., Sheedy J., Mollin A., Cao L. (2019). Effective Delivery of a Microtubule Polymerization Inhibitor Synergizes with Standard Regimens in Models of Pancreatic Ductal Adenocarcinoma. Clin. Cancer Res..

[26] Wang H., Wang L., Erdjument-Bromage H., Vidal M., Tempst P., Jones R.S., Zhang Y. (2004). Role of histone H2A ubiquitination in Polycomb silencing. Nature.

[27] Steele J.C., Torr E.E., Noakes K.L., Kalk E., Moss P.A., Reynolds G.M., Hubscher S.G., van Lohuizen M., Adams D.H., Young L.S. (2006). The polycomb group proteins, BMI-1 and EZH2, are tumour-associated antigens. Br. J. Cancer.

[28] Bhattacharya R., Mustafi S.B., Street M., Dey A., Dwivedi S.K. (2015). Bmi-1: At the crossroads of physiological and pathological biology. Genes. Dis..

[29] Wang R., Fan H., Sun M., Lv Z., Yi W. (2022). Roles of BMI1 in the Initiation, Progression, and Treatment of Hepatocellular Carcinoma. Technol. Cancer Res. Treat..

[30] Wang H., Pan K., Zhang H.K., Weng D.S., Zhou J., Li J.J., Huang W., Song H.F., Chen M.S., Xia J.C. (2008). Increased polycomb-group oncogene Bmi-1 expression correlates with poor prognosis in hepatocellular carcinoma. J. Cancer Res. Clin. Oncol..

[31] Lukacs R.U., Memarzadeh S., Wu H., Witte O.N. (2010). Bmi-1 is a crucial regulator of prostate stem cell self-renewal and malignant transformation. Cell Stem Cell.

[32] Fan L., Xu C., Wang C., Tao J., Ho C., Jiang L., Gui B., Huang S., Evert M., Calvisi D.F. (2012). Bmi1 is required for hepatic progenitor cell expansion and liver tumor development. PLoS ONE.

[33] Wang M.C., Li C.L., Cui J., Jiao M., Wu T., Jing L.I., Nan K.J. (2015). BMI-1, a promising therapeutic target for human cancer. Oncol. Lett..

[34] Bolomsky A., Muller J., Stangelberger K., Lejeune M., Duray E., Breid H., Vrancken L., Pfeiffer C., Hubl W., Willheim M. (2020). The anti-mitotic agents PTC-028 and PTC596 display potent activity in pre-clinical models of multiple myeloma but challenge the role of BMI-1 as an essential tumour gene. Br. J. Haematol..

[35] Flamier A., Abdouh M., Hamam R., Barabino A., Patel N., Gao A., Hanna R., Bernier G. (2020). Off-target effect of the BMI1 inhibitor PTC596 drives epithelial-mesenchymal transition in glioblastoma multiforme. NPJ Precis. Oncol..

[36] Jin X., Kim L.J.Y., Wu Q., Wallace L.C., Prager B.C., Sanvoranart T., Gimple R.C., Wang X., Mack S.C., Miller T.E. (2017). Targeting glioma stem cells through combined BMI1 and EZH2 inhibition. Nat. Med..

[37] Liu L., Andrews L.G., Tollefsbol T.O. (2006). Loss of the human polycomb group protein BMI1 promotes cancer-specific cell death. Oncogene.

[38] Rayess H., Wang M.B., Srivatsan E.S. (2012). Cellular senescence and tumor suppressor gene p16. Int. J. Cancer.

[39] Wu K., Woo S.M., Seo S.U., Kwon T.K. (2021). Inhibition of BMI-1 Induces Apoptosis through Downregulation of DUB3-Mediated Mcl-1 Stabilization. Int. J. Mol. Sci..

[40] Letai A.G. (2008). Diagnosing and exploiting cancer’s addiction to blocks in apoptosis. Nat. Rev. Cancer.

[41] Li J., Gong L.Y., Song L.B., Jiang L.L., Liu L.P., Wu J., Yuan J., Cai J.C., He M., Wang L. (2010). Oncoprotein Bmi-1 renders apoptotic resistance to glioma cells through activation of the IKK-nuclear factor-kappaB Pathway. Am. J. Pathol..

[42] Liu Z.G., Liu L., Xu L.H., Yi W., Tao Y.L., Tu Z.W., Li M.Z., Zeng M.S., Xia Y.F. (2012). Bmi-1 induces radioresistance in MCF-7 mammary carcinoma cells. Oncol. Rep..

[43] Akita N., Okada R., Mukae K., Sugino R.P., Takenobu H., Chikaraishi K., Ochiai H., Yamaguchi Y., Ohira M., Koseki H. (2023). Polycomb group protein BMI1 protects neuroblastoma cells against DNA damage-induced apoptotic cell death. Exp. Cell Res..

[44] Hooks K.B., Audoux J., Fazli H., Lesjean S., Ernault T., Dugot-Senant N., Leste-Lasserre T., Hagedorn M., Rousseau B., Danet C. (2018). New insights into diagnosis and therapeutic options for proliferative hepatoblastoma. Hepatology.

[45] Karlsson M., Zhang C., Mear L., Zhong W., Digre A., Katona B., Sjostedt E., Butler L., Odeberg J., Dusart P. (2021). A single-cell type transcriptomics map of human tissues. Sci. Adv..

[46] Eichenmüller M., Gruner I., Hagl B., Häberle B., Müller-Höcker J., von Schweinitz D., Kappler R. (2009). Blocking the hedgehog pathway inhibits hepatoblastoma growth. Hepatology.

[47] Pfaffl M.W. (2001). A new mathematical model for relative quantification in real-time RT-PCR. Nucleic Acids Res..

[48] Metsalu T., Vilo J. (2015). ClustVis: A web tool for visualizing clustering of multivariate data using Principal Component Analysis and heatmap. Nucleic Acids Res..

[49] Demir S., Kessler T., Hotes A., Haberle B., Hiyama E., Hishiki T., Indersie E., Branchereau S., Vokuhl C., Dorel M. (2025). Mechanistic models position ceritinib as a nuclear integrity disrupting therapy in pediatric liver tumors. J. Exp. Clin. Cancer Res..

[50] Dobin A., Davis C.A., Schlesinger F., Drenkow J., Zaleski C., Jha S., Batut P., Chaisson M., Gingeras T.R. (2013). STAR: Ultrafast universal RNA-seq aligner. Bioinformatics.

[51] Anders S., Pyl P.T., Huber W. (2015). HTSeq—A Python framework to work with high-throughput sequencing data. Bioinformatics.

[52] Love M.I., Huber W., Anders S. (2014). Moderated estimation of fold change and dispersion for RNA-seq data with DESeq2. Genome Biol..

[53] Jung L.A., Demir S., Hotes A., Hiyama E., Hishiki T., Indersie E., Branchereau S., Cairo S., Kappler R. (2025). Targeting HSP90 with Ganetespib to Induce CDK1 Degradation and Promote Cell Death in Hepatoblastoma. Cancers.

